# Response of human peripheral blood monocyte-derived macrophages (PBMM) to demineralized and decellularized bovine bone graft substitutes

**DOI:** 10.1371/journal.pone.0300331

**Published:** 2024-04-18

**Authors:** K. G. Aghila Rani, Ahmed M. Al-Rawi, Ali Al Qabbani, Sausan AlKawas, Mohammad G. Mohammad, A. R. Samsudin

**Affiliations:** 1 Research Institute for Medical and Health Sciences, University of Sharjah, Sharjah, United Arab Emirates; 2 Oral and Craniofacial Health Sciences Department, College of Dental Medicine, University of Sharjah, Sharjah, United Arab Emirates; 3 Department of Medical Laboratory Sciences, College of Health Sciences, University of Sharjah, Sharjah, United Arab Emirates; Kimura Hospital, JAPAN

## Abstract

The performance of apparently biocompatible implanted bovine bone grafts may be compromised by unresolved chronic inflammation, and poor graft incorporation leading to implant failure. Monitoring the intensity and duration of the inflammatory response caused by implanted bone grafts is crucial. In this study, the ability of demineralized (DMB) and decellularized (DCC) bovine bone substitutes in initiating inflammatory responses to peripheral blood monocyte-derived macrophages (PBMMs) was investigated. The response of PBMMs to bone substitutes was evaluated by using both direct and indirect cell culture, reactive oxygen species (ROS) generation, apoptosis, immunophenotyping, and cytokine production. Analysis of DMB and DCC substitutes using scanning electron microscope (SEM) showed a roughened surface with a size ranging between 500 and 750 μm. PBMMs treated with DMB demonstrated cell aggregation and clumping mimicking lipopolysaccharide (LPS) treated PBMMs and a higher proliferation ability (166.93%) compared to control (100%) and DCC treatments (115.64%; p<0.001) at 24h. This was associated with a significantly increased production of intracellular ROS in PBMMs exposed to DMB substitutes than control (3158.5 vs 1715.5; p<0.001) and DCC treatment (2117.5). The bone substitute exposure also caused an increase in percentage apoptosis which was significantly (p<0.0001) higher in both DMB (27.85) and DCC (29.2) treatment than control (19.383). A significant increase in proinflammatory cytokine expression (TNF-α: 3.4 folds; p<0.05) was observed in DMB substitute-treated PBMMs compared to control. Notably, IL-1β mRNA was significantly higher in DMB (21.75 folds; p<0.0001) than control and DCC (5.01 folds). In contrast, DCC substitutes exhibited immunoregulatory effects on PBMMs, as indicated by the expression for CD86, CD206, and HLDR surface markers mimicking IL-4 treatments. In conclusion, DMB excites a higher immunological response compared to DCC suggesting decellularization process of tissues dampen down inflammatory reactions when exposed to PBMM.

## Introduction

Bone grafting is one of the most popular surgical techniques employed for enhancing bone regeneration in dental and orthopedic treatments. [1, 2]. In dental surgeries, the most prevalent use of bone grafting is while using implants to replace the edentulous portion of a missing tooth. Interestingly, one out of four dental implants require bone grafting [3]. The number of procedures involving the repair of bone defects is estimated to grow by 13% annually [4]. Despite this widespread use of bone grafts and substitute materials, limitations persist, raising concern for the end users, especially for allographs and autografts [3]. Several bone graft materials were reported in the literature, including synthetic, autogenous, allogenic, and xenogeneic sources [5]. Xenogenic bone grafts have shown great promise and the most widely used sources are from bovine, porcine, and coral origins [3]. Demineralized bone matrices (DBM) derived from xenogenic sources are approved medical devices used in bone defects with a long track record of clinical use in diverse forms [6]. However, the safe use in clinics is still a challenge. Main challenge of using xenogenous bone grafts is the risk of transmitting various diseases and associated inflammation [7]. Implanting xenogeneic bone grafts into bone defects can trigger innate immune reactions such as causing tissue-resident macrophages to release pro-inflammatory cytokines like TNF-α and IL-1β, resulting in further complications at the surgical site [8]. Although inflammation is crucial for the initial stages of wound healing, excessive or persistent inflammation may adversely interfere with the effectiveness of biomaterial implants and wound healing [9].

Immunogenicity and inflammatory responses against implanted devices are influenced by components of implanted material immunogenic potential, such as the surface topography and physicochemical characteristics and its ability to provoke the production of inflammatory cytokines [10]. The intense initial inflammatory response following implantation of xenografts is driven by proinflammatory cytokines released by immune cells in the implant microenvironment and their levels can play a crucial role in determining the extent of the reaction from biomaterials. Macrophages are important mediators of such inflammatory immune responses and often respond by adverse inflammatory and fibrotic responses to surface chemistry of implanted biomaterials [11], typically related to the foreign body response (FBR) [12]. The phenotypically and functionally categorized M1 (pro-inflammatory) and M2 (anti-inflammatory) macrophages are among the extensively studied subsets [13, 14]. A comprehensive understanding of the phenotypic and functional characteristics of macrophages is critical for effective tissue repair. This understanding will also help for a rational design of biomaterials favoring biomaterial-tissue integration and regeneration. The type, quantity and the conformation of the adsorbed proteins on the surface of the biomaterials, and their interaction with immune cell populations, further determine the cellular-driven inflammatory responses post-implantation of the biomaterials [15]. Likewise, the presence of nucleic acids remnants can mediate a long-term stimulation of immunogenic reactions causing chronic inflammation at the site of xenograft implantation that may become resistant to local treatment. The final sequalae is usually the failure of the bone grafts and rejection of the implanted xenogeneic material. Decellularization methods were used to avoid these side effects [16, 17]. Further, decellularized bone matrix were reported as excellent bio-scaffolds in bone tissue engineering with high biocompatibility properties [18–20]. In the process of decellularization, the immunogenic content of the source tissues is eliminated significantly which includes removal of both cellular and genetic components while retaining the bony tissue architecture and non-cellular matrix [18].

Peripheral blood monocyte-derived macrophages (PBMMs) serve as an attractive *in vitro* model to investigate biocompatibility and inflammatory response parameters of biomaterials. PBMMs enable researchers to evaluate the response of innate immune cells, assess cytokine production, and gain insights into the potential inflammatory reactions induced by the biomaterials. In a previous study, we reported the development of a novel decellularization technique for the production of safe decellularized bovine bone (DCC) substitute, as well as a demineralization technique for creating demineralized bovine bone (DMB) substitutes specifically designed to support bone regeneration [21]. On comparison, DCC demonstrated superior biocompatibility and exhibited promising potential for osteogenesis and bone regeneration both *in vitro* [21] and *in vivo* [22], than DMB substitutes. However, understanding the immune response to these bone substitutes is crucial for assessing their safety and optimizing their long-term performance in bone regeneration applications. Therefore, the present study aims at investigating the *in vitro* immune-compatibility of DMB and DCC substitutes using peripheral blood monocyte-derived macrophages (PBMM). A deeper understanding of the immune responses and inflammatory parameters associated with these graft substitutes would contribute valuable insights for improving the safety and efficacy and improving the success rates for future utility in dental and orthopedic treatments.

## Materials and methods

### Preparation of decellularized and demineralized bovine bone substitutes

The DMB and DCC bovine bone samples were prepared according to a previously reported standard procedures [21]. Cancellous bone grafts were prepared from bovine femoral heads that has been stored at -80°C until the time of bone substitutes preparation. The soft tissues were removed, and the bone samples were sectioned using bone cutting bandsaw (JG210 Bone Cutting machine, Shandong, China) into cubes of 1 cm in size. Following washing in running tap water and water jet spray washing (high-pressure water-jet spray with pressure not exceeding 160 psi), the bone cubes were suspended in sterile distilled water and centrifuged for 24h in mild rotation (300 g) to remove debris and red blood cells. The cleaned bone cubes were divided into two groups for demineralization and decellularization treatments [21]. At the end of the treatment procedures, both the DMB and DCC scaffolds were crushed into granules using an IKA A10 S9 bone grinder (Staufen, Germany). DMB and DCC bone granules/substitutes were then subjected to terminal gamma radiation sterilization at 25kGy and then stored at room temperature until use. The particle sizes and morphology were validated using SEM (VEGA3 XM-TESCAN, Czech Republic). Briefly, the prepared DMB and DCC substitutes were placed on Aluminum stubs, air dried, coated with Gold-Palladium and observed under SEM.

### Ethics statement

Blood donors had signed an informed written consent form approved by the University of Sharjah Research Ethics Committee (REC-22-04-20-05).

### Isolation and enrichment of human peripheral blood monocytic macrophages (PBMMs)

The Isolation and enrichment of PBMMs was performed, as previously described [23, 24]. Blood was collected from 4–6 healthy donors into 10 mL blood collection tubes containing EDTA. Samples from donors were pooled to maintain homogeneity among different experiments and to avoid any individual-based variations [23]. PBMCs was enriched by overlaying 12.5 mL of pooled blood onto 10 mL of Histopaque-1,077 (Sigma-Aldrich, St. Louis, MO), followed by centrifugation at 400 g for 25 min at room temperature with the brakes off PBMCs in the interface were carefully pipetted and then washed with phosphate-buffered saline (PBS). Cells pellets then resuspended in one mL of complete RPMI-1640 (supplemented with 10% FBS (Sigma, USA) and 1% penicillin/streptomycin). Viable cells were determined using Trypan blue vital dye. PBMCs were seeded at specified cell densities for each experimental set up as mentioned below and cultured in complete RPMI-1640 media for 24h. On the following day, floating non-monocytic cells were removed by gentle aspiration of the supernatants followed by washing with pre-warmed PBS. Complete RPMI media was then added to the attached monocytic cell cultures which were then incubated for 8 days. DCC and DMB bovine bone substitutes were added to the PBMMs at a concentration of 1mg/mL.

### Cell viability

Cell viability assay was performed using a XTT cell viability kit (Sigma, USA). Cells were seeded into 96-well tissue culture plates at a density of 5×10^4^ cells per 200μL of RPMI-1640 media supplemented with 10% fetal bovine serum (FBS; Sigma) and 1% penicillin/streptomycin (Sigma-Aldrich, St. Louis, MO). Images were taken to examine differences in cell shape and structure among the different experimental groups including untreated control cells, cells treated with LPS, IL-4, DMB and DCC substitutes by direct and indirect methods on day 8 of culture. A total of three separate repeats were carried out for each experimental group (donor number = 6). Viability studies were performed by both direct and indirect methods as recommended in the ISO 10993–5:2009 Part 5 standard [25]. The direct method involves assessment of how surface characteristics of the bone substitutes, such as roughness, porosity, and viscosity, may influence viability and survival of cells. Whereas indirect method entails the preparation of extracts from bone substitutes, which are subsequently incubated with cells, focusing solely on evaluating the impact of substances, including potential toxins, released into the culture medium. For the direct method, DCC and DMB bone substitutes were suspended in a complete culture medium at a concentration of 1mg/mL, introduced into the cells, and subjected to proliferation assay for days 1, 2, 3 and 8. For the indirect method, the bone substitutes were suspended in a complete culture medium at a concentration of 1mg/mL and incubated overnight for one day in a 5% CO_2_ incubator and the supernatants were collected and introduced to the cells. At the end of the experiment, XTT reagent was added as recommended by the manufacturer, incubated for 4h and absorbance was measured at 450 nm using a spectrophotometer (Biotek, USA).

### Apoptosis assay

PBMMs were cultured at a density of 5x10^6^ cells per mL per well in 6-well cell culture plates and incubated in 5% CO_2_ incubator (Binder CO_2_ incubator, Germany) at 37°C for one day. The DCC and DMB substitutes were added to PBMMs at a final concentration of 1mg/mL. Cocultures of PBMMs and substitutes were incubated for 1 day and 8 days at 37°C and 5% CO_2_. At the end of the incubation, cells were harvested using a cell scraper in ice cold conditions, centrifuged at 400 g for 5 min, and resuspended in 500 μl of 1x binding buffer. AnnexinV-FITC (5 μl/sample) (Abcam, USA) was added to the cells and incubated for 10 min in dark at room temperature (25°C) followed by the addition of 5 μl PI staining solution at a concentration of 50 μg/mL (Abcam, USA). Cells were further incubated for 5 min and 10,000 events were acquired using BD FACS Aria.

### ROS production assay

For the total ROS assay, PBMMs were seeded at a density of 5x10^6^ cells per mL per well in 6 well culture plates in complete RPMI media. Cells were allowed to attach for 24h. Cells were then washed with warm PBS and replenished with complete RPMI-1640 alone (control) or media containing the DMB/DCC substitutes at 1mg/mL concentration and incubated further for a period of 1 and 8 days. At the end of the experiment period, ROS assay stain (1x) (Thermofisher, USA) was introduced to cells and incubated further for 60 minutes at 37°C in a 5% CO_2_ incubator. Cells were further harvested using a cell scraper and 10,000 events were acquired using a flow cytometer (BD FACS Aria).

### Phenotypic assessment of PBMMs

For phenotypic characterization of PBMMs, PBMMs were enriched and seeded at a density of 5x10^6^ cells per well in 6 well culture plates as mentioned above. PBMM cultures grown in complete RPMI-1640 were considered as a negative control. PBMMs treated with LPS or IL-4 at concentrations of 100 and 15 ng/mL respectively, served as the positive controls of proinflammatory and anti-inflammatory activation of macrophages. Another PBMMs set were treated with DMB or DCC substitutes (designated as the test groups) at a concentration of 1 mg/mL. Following these treatments, the cells were incubated for a duration of 8 days. Staining with antibodies for flow cytometry analyses performed as per previously reported [23]. In brief, plates containing cultures were placed on ice to facilitate the detachment of PBMMs, cells were scraped and collected into 15 mL falcon tubes, followed by centrifugation at 250 x g for 7 min at 4°C. Cell pellets were re-suspended in 500μL of staining washing buffer (SWB), containing 1% FBS and 1 μM EDTA in PBS. The following antibody panel was used for staining macrophages: CD14, CD16, CD86, CD206, and HLA-DR (all obtained from BD, United States). The details of the antibodies used is given in S1 Table. Macrophages were incubated with antibodies master mix for 30 minutes on ice, in dark, in SWB. Cells were washed with SWB once followed by fixation fixation/permeabilization solution (BD, United States). After a single wash, the pellets were re-suspended in 300μL SWB. Stained PBMMs were then analyzed using FACS Aria flow cytometer, and data were analyzed using FlowJo v10 software (BD, United States).

### Real-time PCR analysis of cytokines expression in PBMMs treated with DMB and DCC substitutes

The effect of DMB and DCC substitutes on the expression of TNF-α, IL1-β, CD80, CD206 and IL-10 genes by PBMMs was evaluated by real time PCR. LPS and IL-4 were used as control for the validation of pro-inflammatory and anti-inflammatory cytokine release. Total RNA was extracted using RNA Minikit (Qiagen, USA). The concentration of RNA was measured using Nano-drop ND1000 (Thermo Scientific, USA). First-strand cDNA was synthesized by reverse transcriptase using the High Script cDNA synthesis kit (Qiagen). The expression of the genes was quantified by real-time RT-PCR analysis using 5X FIREPOL SYBR Green master mix (Solisbiodyne, USA). GAPDH served as an internal control in the reaction. Gene-specific primers used in this study are summarized in Table 1.

**Table 1:** Primers used in qPCR.

*Genes*	Primer sequences
*TNF-α*	FP: CAAGGACAGCAGAGGACCAG RP: TCCTTTCCAGGGGAGAGAGG
*IL1-β*	FP: AACCTCTTCGAGGCACAAGG RP: AGCCATCATTTCACTGGCGA
*CD80*	FP: GGGAAATGTCGCCTCTCTGA RP: TGGATGGTGATGTTCCCTGC
*CD206*	FP: GCCTCGTTGTTTTGCGTCTT RP: GAGAACAGCACCCGGAATGA
*IL-10*	FP: GGTCGTGTGCTTGGAGGAAG RP: AGCAGGTGACTCCCACTGTA
*GAPDH*	FP: CCACTCCTCCACCTTTGACG RP: CCACCACCCTGTTGCTGTAG

### Statistical analysis

Statistical analysis was carried out using GraphPad Prism software (version 9.3.1). Differences between the study groups comparing the effect of DMB and DCC treatments in comparison with untreated control over different assays were calculated by mixed ANOVA (Tukey’s multiple comparisons test). P< 0.05 was considered statistically significant.

## Results

### Size analysis of DMB and DCC substitutes using scanning electron microscopy (SEM)

**Fig 1:**
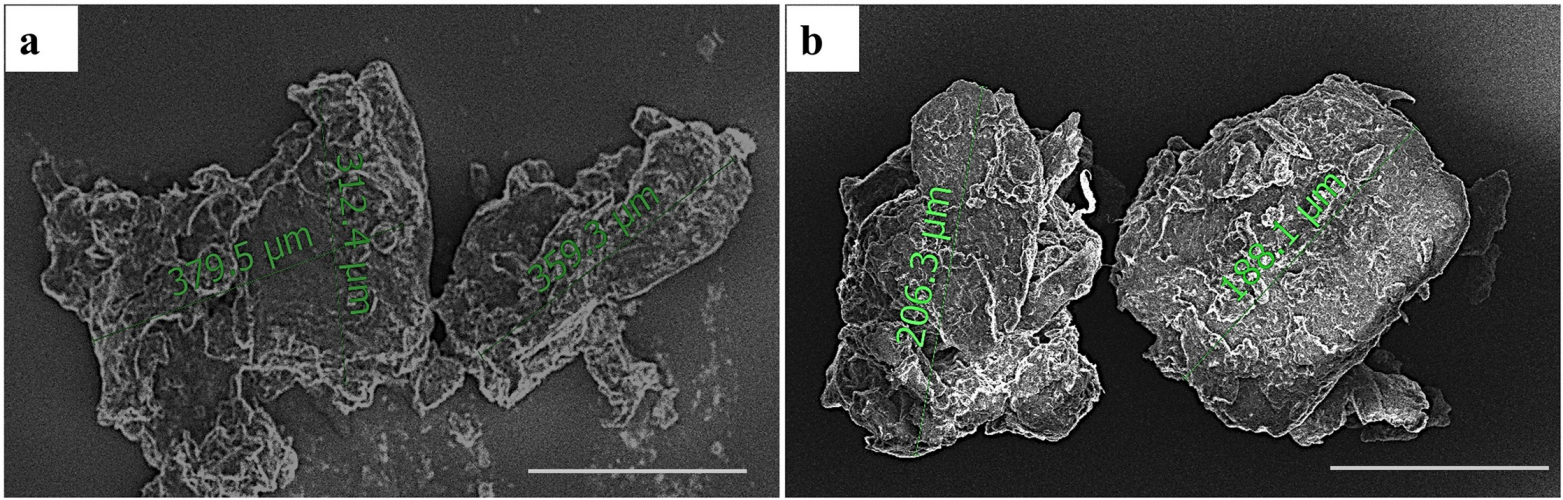
Scanning Electron Microscope image analysis of (a) demineralized bone (DMB) and (b) decellularized bone (DCC) substitutes. The DMB and DCC substitutes ranged in size from 500–750μm. Scale bar = 400 μm.

The SEM analysis of the processed DMB and DCC substitutes under magnification (300X) and scale of measurement of 200 μm revealed a mean size range of 500 to 750 μm, as shown in Fig 1a and 1b. Both substitutes presented a roughened surface with low porosity.

### PBMMs aggregation and clumping in DMB substitute treatment mimic LPS-induced effects

**Fig 2:**
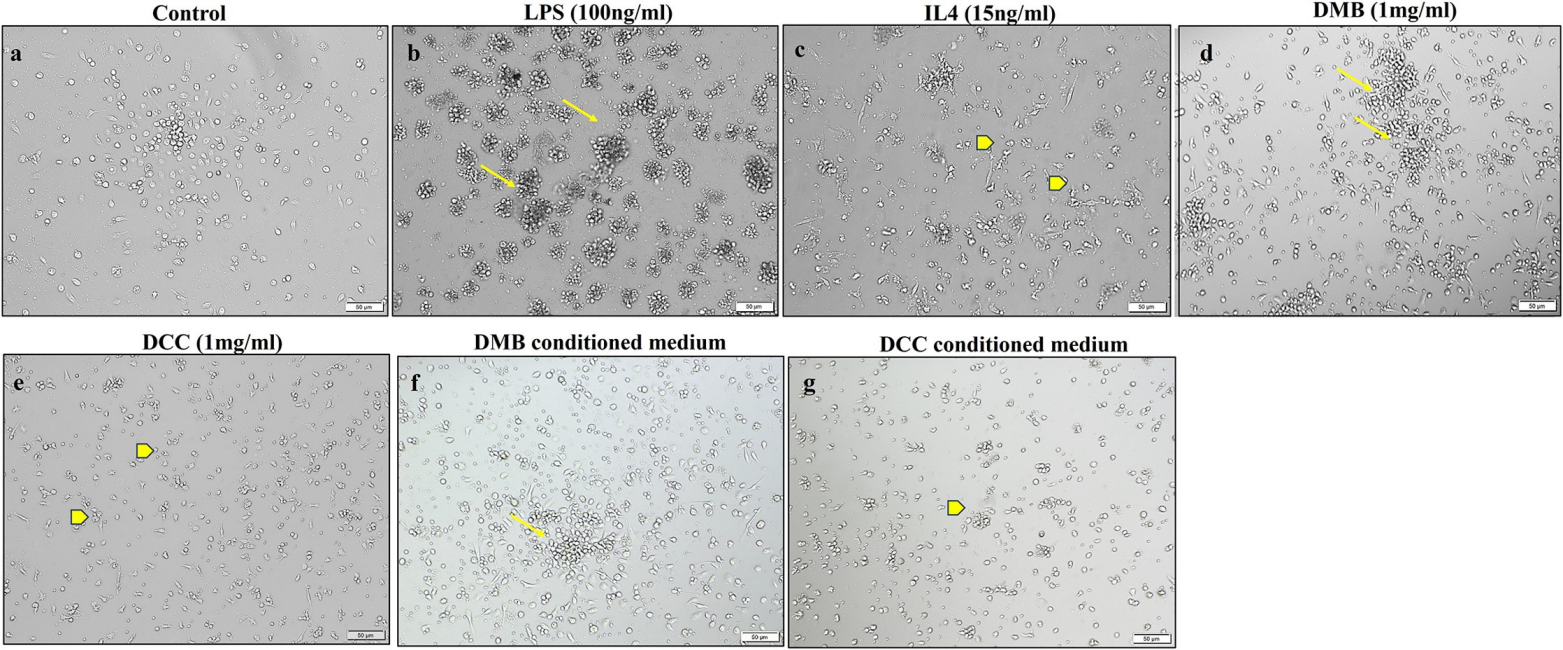
Morphology of peripheral blood monocyte-derived macrophages (PBMM) exposed to DMB and DCC. Representative images showing the morphology of PBMMs grown in (a-e) direct and (f, g) indirect presence of DMB and DCC substitutes in comparison to untreated, LPS (b) and IL-4 treated cells (c) on day 8 of culture. Arrows indicate proliferation and clumping of cells (b, d & f). Arrow heads indicate DCC treated PBMMs exhibiting a spread-out morphology similar to IL-4 treatments. (c, e & g). Scale bar = 50 μm.

PBMMs response to DMB and DCC substitutes at day 8 post- direct and indirect exposure along with the LPS and IL-4 treatment controls was visualized using light microscopy. Qualitative evaluation of the adherent population showed an increasing tendency for cell aggregation and clumping in DMB substitute treated cells similar to that observed in LPS-treated PBMMs as compared to DCC substitutes by both direct and indirect methods (Fig 2). While DCC treated PBMMs exhibited a spread-out morphology similar to IL-4 treatment.

### DMB substitute treatment increased PBMMs proliferation

**Fig 3:**
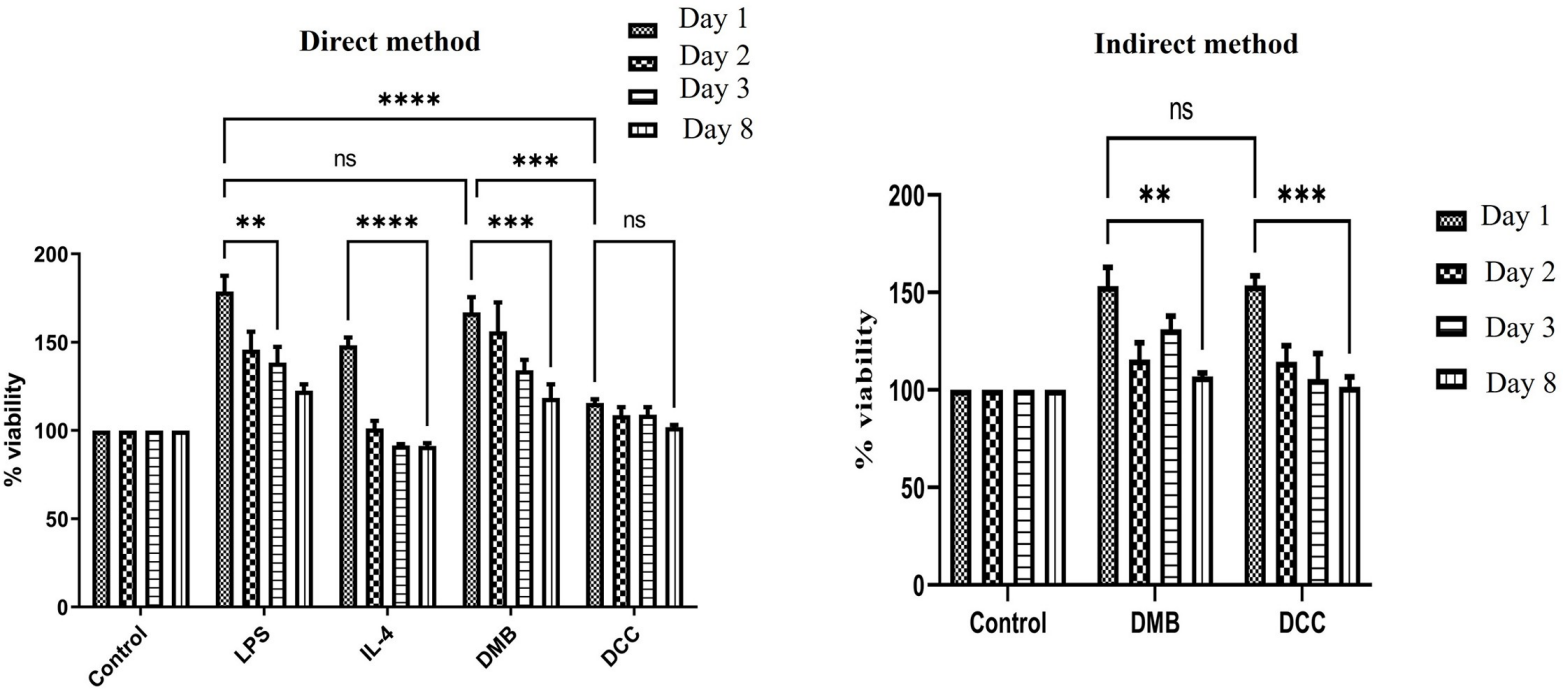
Cell proliferation assay of PBMCs grown in direct (a) or indirect (b) contact with DCC and DMB substitutes in comparison to LPS (100ng/mL), IL-4 (15ng/mL) and cells grown in normal tissue culture medium. *p<0.01, **p<0.001, and ns—non-significant is indicated. Error bars represent standard error of means of three independent measurements.

The cell viability of PBMMs post direct and indirect exposure to DMB and DCC substitutes or their extracts revealed comparable trends. In all the treatment conditions, proliferation of PBMMs was observed as early as the first day followed by decline in viability. In comparison to the untreated controls, a significant decline in cell proliferation was observed by day 8 in both LPS and IL 4 treatment groups (p<0.0001; Fig 3a and 3b). DMB substitute treatment showed the same pattern as LPS at day 1 (Fig 3a). A decline in cell proliferation was observed for DMB substitute treatment by day 8 in both direct and indirect methods (p<0.001; direct method and p<0.01; indirect method). This finding corresponds with the observation that DMB treatment exhibited higher levels of proliferation (p < 0.001) compared to DCC treatments in the direct method. However, no significant difference was found between the two methods in the indirect treatment.

For DCC substitutes, cell proliferation rate was significantly lower post DCC substitute treatment compared to LPS treatment (p<0.0001) as early as day 1. Cell viability remained comparable to the untreated control group (cells grown in complete culture medium) and consistent across different treatment time points, showing no significant variations in the direct-treatment method, whereas in indirect treatments of DCC substitutes, a significant decline in viability (p<0.01; day 8 vs day 1) was observed.

### The effect of DMB and DCC substitutes on PBMMs oxidative stress and cellular apoptosis

**Fig 4:**
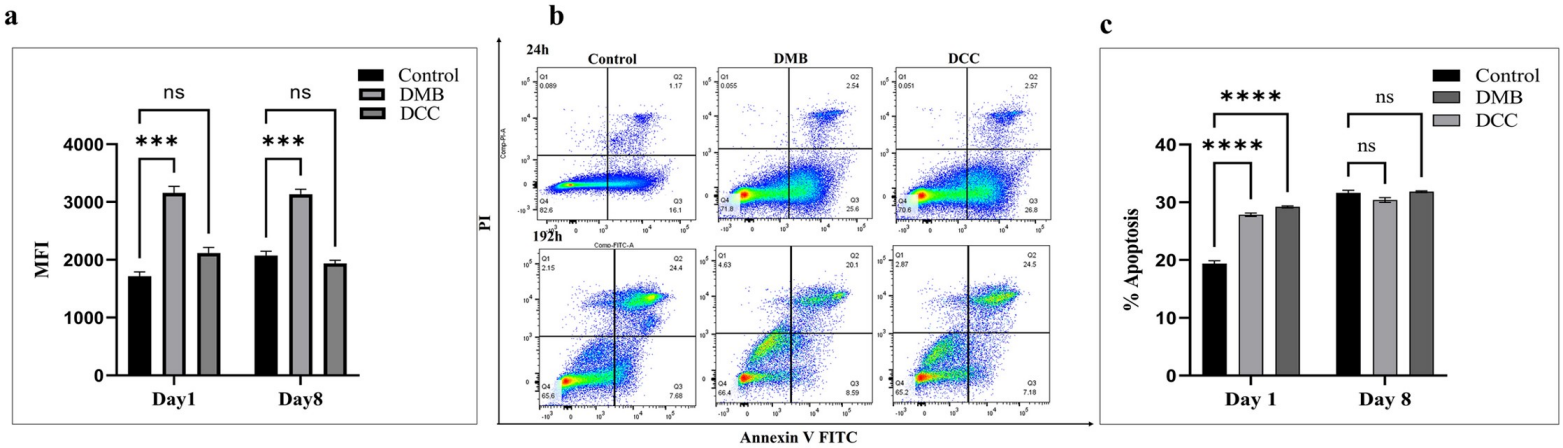
Reactive oxygen species (ROS) generation and cellular apoptosis in PBMMs post-exposure to DMB and DCC substitutes in comparison to untreated control cells. (a) A significant increase in ROS generation was observed for cells grown in the presence of DMB substitutes at day 1 and day 8 when compared to control cells (***p < 0.001). (b) FITC-Annexin V and propidium iodide (PI) cell apoptotic assay of PBMMs. Flow cytometry dot plots showing cellular apoptosis in PBMMs grown for day 1 and day 8 in the presence of 1mg/mL of DMB and DCC substitutes in comparison to control cells grown in normal tissue culture medium. (c) Percentage of both early (Q3) and late (Q2) apoptosis in PBMMs. ****p < 0.0001, and ns–no significance is indicated. Error bars represent standard error of means of three independent experiments.

The intracellular generation of ROS in PBMMs post-exposure to DMB and DCC substitutes was determined after day 1 and day 8 of culture (S1 Fig). PBMMs treated with DMB substitute showed significant increase (p<0.001) in intracellular ROS levels at both day 1 and day 8 of culture compared to untreated control cells (Fig 4a), while there was no significant change in ROS level in PBMMs treated with DCC substitutes as compared to untreated control PBMMs at both time points (Fig 4a). In the case of cellular apoptosis, both DMB and DCC substitute treatment demonstrated a significant increase in percentage apoptosis compared to untreated control cells at day 1 (p<0.0001). However, by day 8, the percentage of apoptosis in PBMMs was comparable to control cells in both treatment groups (Fig 4b and 4c).

### The effect of DMB and DCC substitutes on phenotypic markers of PBMMs

The activation and polarization of PBMMs post-exposure to DMB and DCC for 8 days involved the evaluation of CD14, CD16, CD206, CD86, and HLA-DR surface markers. The M1 subset was induced using LPS while the M2 subset was induced by IL-4, and both served as a positive control in the experiment. The data were presented based on the MFI on acquired gated events of specific markers as indicated in the gating strategy (S2 Fig).

**Fig 5:**
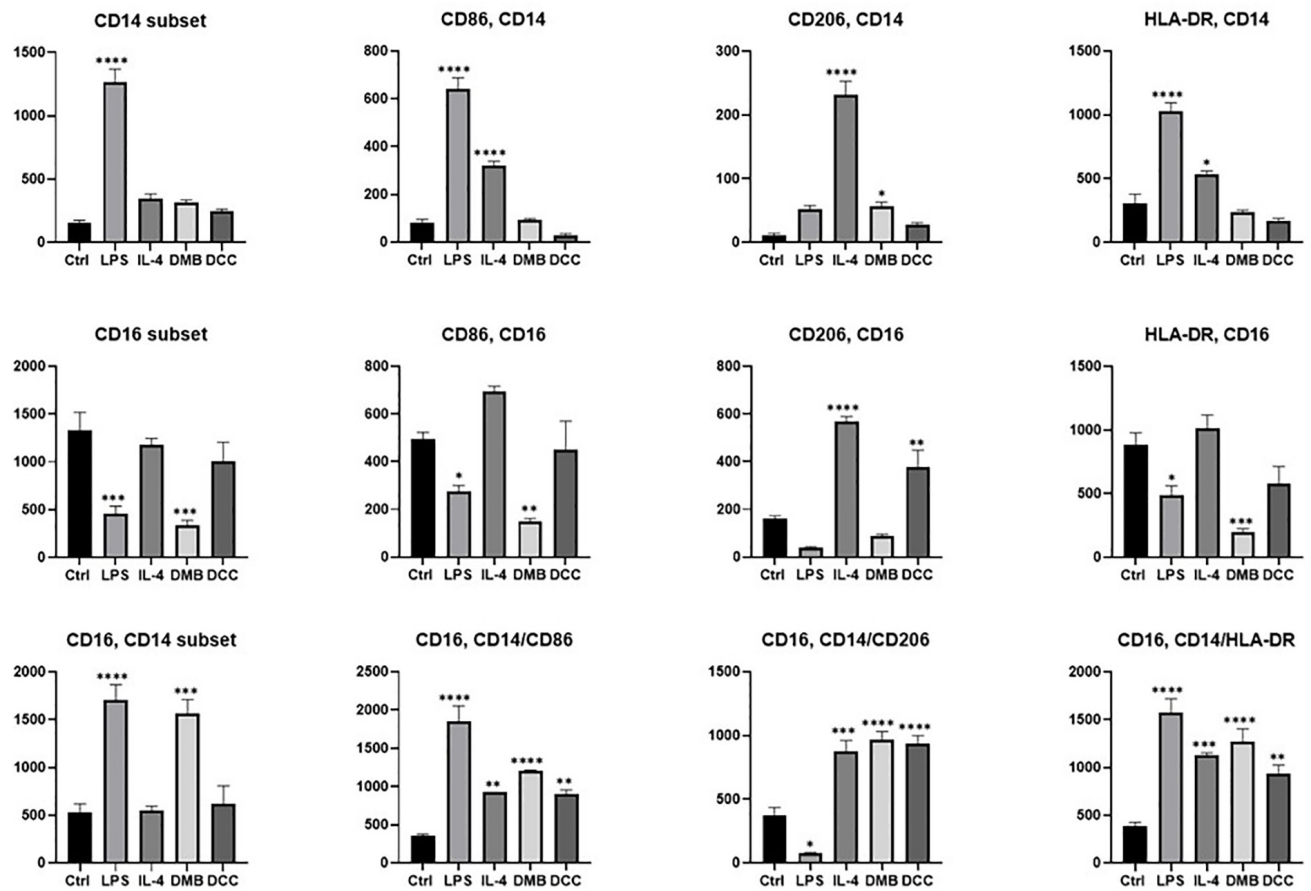
Phenotypic analysis of PBMMs polarization after treatment with LPS, IL-4, DMB and DCC substitutes for 8 days. Phenotypic characterization of macrophages after treatment with bone substitutes was investigated by examining the differential expression of macrophage-related markers like CD14, CD16, CD86, and CD206 based on MFI on gated events. Phenotypic characterization of macrophages after treatment with bone substitutes was investigated by examining the differential expression of macrophage-related markers like CD14, CD16, CD86, and CD206 based on MFI acquired from gated events. One-way ANOVA was used to analyze the data, and the findings were presented as mean + standard error of means. * p<0.05, **< p0.01, *** p<0.001, and **** p<0.0001 is indicated. Error bars represent standard error of means of three independent experiments.

Macrophage polarization into classical, non-classical, and intermediate groups was based on the CD14^+^ CD16, CD14 CD16^+^, and CD14^+^ CD16^+^ gating (S2 Fig). Subsequently, the expressions of CD86, CD206 and HLA-DR were analyzed within each group of CD14^+^, CD16^+^ and CD14^+^CD16^+^ populations, as illustrated in Fig 5. PBMMs treated with LPS demonstrated a significantly higher population in the CD14^+^ subset (p<0.0001), accompanied by a notable increase in the activation markers CD14^+^CD86^+^ (p<0.0001) and CD14^+^HLA-DR^+^ (p<0.0001). In contrast, IL-4 treatments showed higher CD16^+^ population which exhibited high levels of CD16^+^CD86^+^ and CD16^+^CD206^+^ (p<0.0001). Both DMB and DCC treatments did not increase the expression of CD14^+^CD86^+^, CD14^+^CD206^+^ and CD14^+^HLA-DR^+^ macrophages. In CD16^+^ macrophages, DCC substitutes increased the expression of CD16^+^CD86^+^, CD16^+^CD206^+^ (p<0.01) and CD16^+^HLA-DR^+^. DMB treatments significantly increased the intermediate PBMMs population (CD14^+^, CD16^+^ double positive population), (p<0.001). Both DMB and DCC upregulated CD14^+^CD16^+^CD86^+^, CD14^+^CD16^+^CD206^+^ and CD14^+^CD16^+^HLA-DR^+^ on intermediate macrophages. Intermediate macrophages demonstrated a similar profile for most of the surface markers in LPS and DMB treated PBMMs whereas IL-4 and DCC treated PBMMs exhibited a comparable expression profile in all the surface markers.

### Pro and anti-inflammatory cytokines expression following DMB and DCC substitute treatments

**Fig 6:**
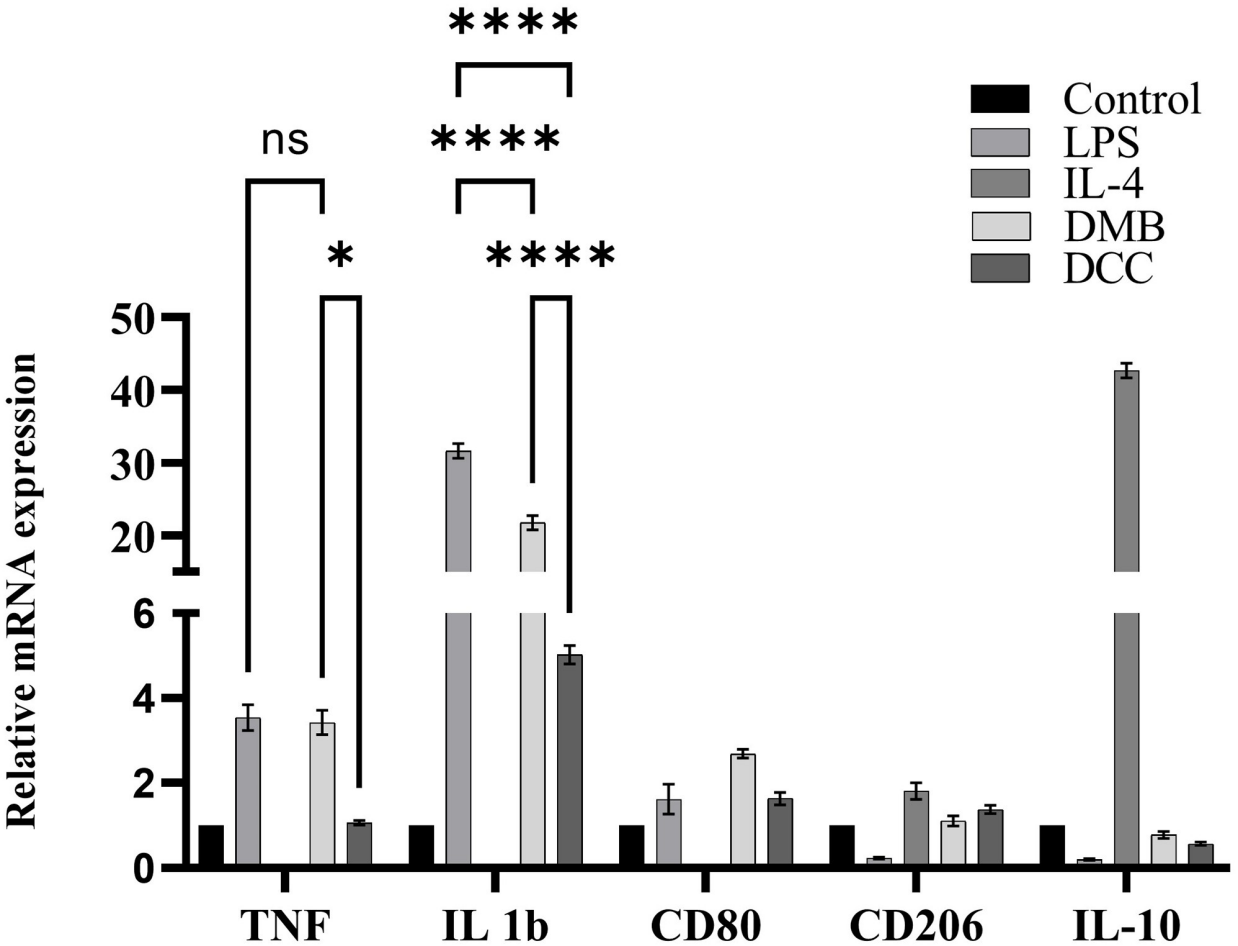
The expression of TNF-α, IL1-β, and IL-10 cytokines and CD80 and CD206 markers in PBMMs treated with 1mg/mL of DMB or DCC substitutes in comparison to LPS and IL-4 treatments, or untreated control cells. The data was normalized with cells grown in a normal tissue culture medium and GAPDH served as the internal housekeeping control gene. *p<0.05, and ****p<0.0001 and ns-non-significant are indicated. Error bars represent standard error of means of three independent experiments.

Fig 6 presents the results of real-time PCR analysis, showing the expression of cytokines following treatments with DMB and DCC substitutes, along with untreated controls, LPS and IL-4 treatments. PBMMs treated with LPS exhibited a significant increase in mRNA for inflammatory cytokines TNF-α and IL-1β and the activation marker CD 80 when compared to untreated control cells, whereas their levels declined for CD206 and the anti-inflammatory IL-10 cytokine. DCC group showed a significant decline in TNF-α (p<0.05) and IL-1β (p<0.0001) levels compared to LPS treatments, however, the levels of CD80, CD206, and IL-10 was not found to be significantly varied. In DMB group, the levels of IL-1β were found to significantly declined (p<0.0001) whereas TNF-α levels remain comparable to LPS. DMB group showed a significantly higher expression levels for TNF-α (p<0.05) and IL-1β (p<0.0001) cytokines compared to DCC group. However, the levels of CD80, CD206, and IL-10 mRNA levels were not observed to be significantly different among DCC and DMB treatments.

A comprehensive summary of cellular characteristics and responses in PBMMs treated with DMB and DCC substitutes in comparison to untreated control cells is shown in Table 2.

**Table 2:** Summary of cellular characteristics and responses in PBMMs treated with DMB and DCC substitutes.

Cellular responses	DMB	DCC
Cell aggregation	High	Absent
Cell proliferation	High	Similar to untreated control
ROS production	High	Similar to untreated control
Apoptosis	High	Non-significant
Pro-inflammatory cytokines	High	Low
Macrophage polarization	M1	M2

## Discussion

The utilization of DCC grafts presents a promising avenue in bone tissue engineering. These scaffolds offer several advantages, including biocompatibility, osteoconductive properties and a porous structure that allows for cell infiltration and vascularization [26]. The acellular matrix in DCC scaffolds is an additional advantage that favors less risk of transplantation of nucleic acids into future recipients, thus rendering lower risk of immunogenicity. However, before these grafts can be safely employed in clinical settings, it is crucial to thoroughly evaluate their immune compatibility. The interaction between the graft and the host’s immune system can profoundly influence the success of the regenerative process and the long-term viability of the implanted tissue. By conducting comprehensive immuno-compatibility assessments, valuable insights could be gained into the graft’s potential immunogenicity, possible delayed adverse reactions, and develop strategies to mitigate any risks. This study has paved the way for safer and more successful clinical applications of DCC grafts in bone tissue regeneration when its processing techniques was able to produce a bone substitute that meets the recommended acceptable level of residual nucleic acid content in biological tissue scaffolds for safe transplantation [16, 21]. While the DMB demonstrated partial elimination of cellular nuclear material, DCC achieved a more comprehensive removal of cells and genetic material [21], which render it both bio and immuno-compatible.

In the field of biomaterial testing, PBMCs have emerged as a valuable tool for evaluating immune compatibility [27]. The exposure of PBMMs to the biomaterial of interest allows researchers to mimic the *in vivo* immune micro-environment and monitor cellular responses such as cell proliferation, cytokine production, and macrophages phenotypic changes [28, 29]. In the current study, the qualitative evaluation of the adherent population by microscopy showed a tendency for increased cell aggregation and clumping in both direct and indirect DMB substitute treated cells compared to DCC treatment, resembling LPS treatments. The microscopic appearance of the proliferating clumps of cells indicated a possible differentiation of monocytic cells to M1 macrophage lineage post 8 days of DMB substitute treatment. In a previous study, David BK *et al* observed a similar initial heightened immune response during the early phase of healing following implantation of demineralized freeze-dried bovine cortical bones (DFDBCBM), followed by a normal tissue response post 4 weeks of implantation [21]. On the other hand, the presence of spindle-shaped cells in DCC substitute treatment group indicated the potential differentiation of monocytic cells into M2 lineage macrophages [30]. These findings support a previous *in vivo* study by You *et al*., in which super critical CO_2_-based decellularization technique was employed to process bovine bone grafts that was then subjected to cellular and molecular immunological response study in mice. Compared with the fresh bone group, the immune responses of decellularized group were reported to be significantly reduced [31].

The decline in PBMM proliferation observed after LPS treatments from day 1 to day 3 in the current study suggested a time-dependent response of the macrophages. Initially, LPS stimulation triggered an immediate activation of PBMMs, leading to increased proliferation and immune response. However, as time progressed, a decline in cell proliferation was observed, plausibly due to various processes such as oxidative stress, activation-induced cell death or the establishment of negative feedback mechanisms to regulate the immune responses [32].

Oxidative stress plays a pivotal role in initiating inflammatory responses. The increased production of reactive oxygen species (ROS), resulting in oxidative stress and inflammation is accountable for the toxicity of engineered biomaterials [33, 34]. The detrimental effects of increased ROS production and oxidative stress in response to biomaterials implantation can result in cellular dysfunction, DNA damage and activation of pro-inflammatory pathways [35]. Comparison studies of ROS levels in the present study post the first and the eighth day of PBMM culture provided insights into the potential differential effects of the two bone substitutes on intracellular ROS production. This differential effect of DMB and DCC substitutes on ROS release in PBMMs have important implications for their potential biological effects and safety considerations. The lack of significant changes in ROS levels in DCC substitute-treated cells suggested a relatively lower risk of oxidative stress-related cellular damage and the aggravation of subsequent inflammatory processes. The significant increase in ROS release observed in DMB substitute-treated cells highlights the potential for enhanced oxidative stress and associated adverse effects on cellular functions that include recruiting polymorphonuclear cells and monocytes into the sites of implantation, which may further exacerbates the inflammatory responses [36]. This oxidative stress subsequently triggered cellular apoptosis in DMB treated cells. Although the day 1 of exposure displayed a short-term effect showing both treatment effectively induced apoptosis in PBMMs, interestingly, as the study progressed to day 8, the percentage of apoptosis in PBMMs treated with either DMB or DCC substitutes became comparable to that of control cells. This could be attributed to cellular repair mechanisms that may contribute to the adaptation of the foreign material by restoring of cell viability and the resolution of the apoptotic response upon long-term exposure to foreign materials [37, 38]. In a prior investigation by Niska *et al*., it was revealed that exposing HT22 cells to CuO nanoparticles led to a similar elevation in ROS production, triggering oxidative stress and subsequent cellular apoptosis [34].

To further address assess how surface properties of substitutes influence the inflammatory response of PBMMs *in vitro*, the levels of cell surface protein expression and cytokine production were measured after eight days of culturing PBMMs with the DMB and DCC substitutes. The phenotypic assessment of PBMMs after exposure to DMB and DCC substitutes for 8 days revealed important insights into their immune response pattern by impacting macrophage polarization and activation states. The intermediate macrophages showed marker expression patterns mostly consistent with LPS [39] and DMB treatment, whereas IL-4 and DCC treatments displayed similar expression profiles across all surface markers, suggesting an immunoregulatory behavior of DCC on PBMMs.

Inflammation is primarily driven by activated macrophages, which secrete pro-osteolytic mediators such as TNF-α, IL-1β, IL-6, and IL-8, resulting in monocyte recruitment, suppression of osteoblastic differentiation and induction of osteoclastogenesis [40]. Activation of macrophages thus plays a crucial role in peri-implant osteolysis. The production of inflammatory cytokines by PBMMs in response to biomaterials plays a vital role in mediating the host response that can significantly impact tissue integration and regeneration. As a result, evaluating the generation of inflammatory cytokines by PBMM when exposed to biomaterials is a critical aspect of assessing biocompatibility. In the current study, DMB substitute treatment resulted in an increased expression of TNF-α and IL-1β cytokines. A previous study examined the inflammatory reaction caused by three different commercially available demineralized matrices (DBM) using a murine air pouch model, highlighting the importance of considering both efficacy and safety of materials and methods used for their processing when selecting a bone grafting method [41]. At the same time demineralized bone matrix (DBM) generated by yet another method exhibited a lower inflammation-induced expression of inflammatory mediators in murine cell-based bioassays [42]. However, current understanding allows modification of biomaterial physico-chemical design to support biomaterial-induced macrophage polarization for tissue regeneration [43]. In an *in vitro* pilot study by Panahipour *et al*., it was found that DBM demonstrated anti-inflammatory activity by reducing the expression of IL-1β and IL-6 in RAW 264.7 macrophage cells, suggesting a potential modulation of the inflammatory responses [42]. Such conflicting results may be attributed to differences in the methodology of the studies, particularly in terms of the graft processing techniques utilized, which produced variable physico-chemical and topographical characteristics that elicit varying host tissue responses besides the likely presence of residual native cells within the grafts that may evoke the inflammatory responses.

In conclusion, the evaluation of PBMM responses in biomaterial testing is crucial for understanding the host response to bovine bone scaffolds and other biomaterials. By assessing parameters such as cell proliferation, viability, oxidative stress and inflammatory cytokine production, essential information on the biocompatibility and potential immunomodulatory effects of these scaffolds in the future clinical setting could be predicted and the necessary modifications could be implemented, allowing reduction in number of animal studies and safer clinical trials.

In the current study, the observation that the direct exposure of DMB substitutes caused aggregation of macrophages, i.e., the balance with anti-inflammatory M2 was disrupted and resulted in the upregulated mRNA levels of relevant proinflammatory cytokines. The results of this study also suggest that decellularized bovine grafts exhibit improved biocompatibility and reduced oxidative stress, leading to decreased production of pro-inflammatory cytokines. This could be attributed to the gentle processing protocol employed for the development of DCC substitutes, which resulted in an enhanced acellular material while preserving the structural and functional components of its extracellular matrix. Additionally, the absence of cellular and genetic materials in the DCC substitutes likely contributed to the suppression of initial monocyte activation into proinflammatory, M1 lineage macrophages. Studies investigating the immunological reactions of decellularized bovine bone grafts using PBMMs as a model are limited, and to the best of our knowledge, the current study represents a pioneering investigation in this area, providing novel insights in this field with therapeutic potential.

## Supporting information

S1 Fig Gating strategy for reactive oxygen species (ROS) determination by flow cytometry analysis. (a). Peripheral Blood Monocyte-derived Macrophages (PBMM) were gated based on forward and side scatter, (b) Doublet cells were excluded. (c) Representative histogram of reactive oxygen species expression in cells (d) analysis of results comparing the median fluorescent intensity (MFI) acquired from gated events among the study groups, (e) Representative histograms of ROS expression among unstained, untreated control, DMB and DCC treatments.
(TIF)

S2 Fig Gating strategy for flow cytometry analysis. (a) Peripheral Blood Monocyte-derived Macrophages (PBMM) were gated based on forward and side scatter. (b) Doublet cells were excluded. (c) CD14 and CD16 subsets of macrophages were gated. (d) The different subpopulations of macrophages were determined based on the CD86, HLADR and CD206 gating followed by (e) analysis of results.
(TIF)

S1 Table Details of antibodies used in flow cytometry analysis. (DOCX)
