# Supplementary figures and images for: Response of human peripheral blood monocyte-derived macrophages (PBMM) to demineralized and decellularized bovine bone graft substitutes
Source: PLoS One. 2024 Apr 18;19(4):e0300331. doi: 10.1371/journal.pone.0300331 (PMC11025794; doi:10.1371/journal.pone.0300331)

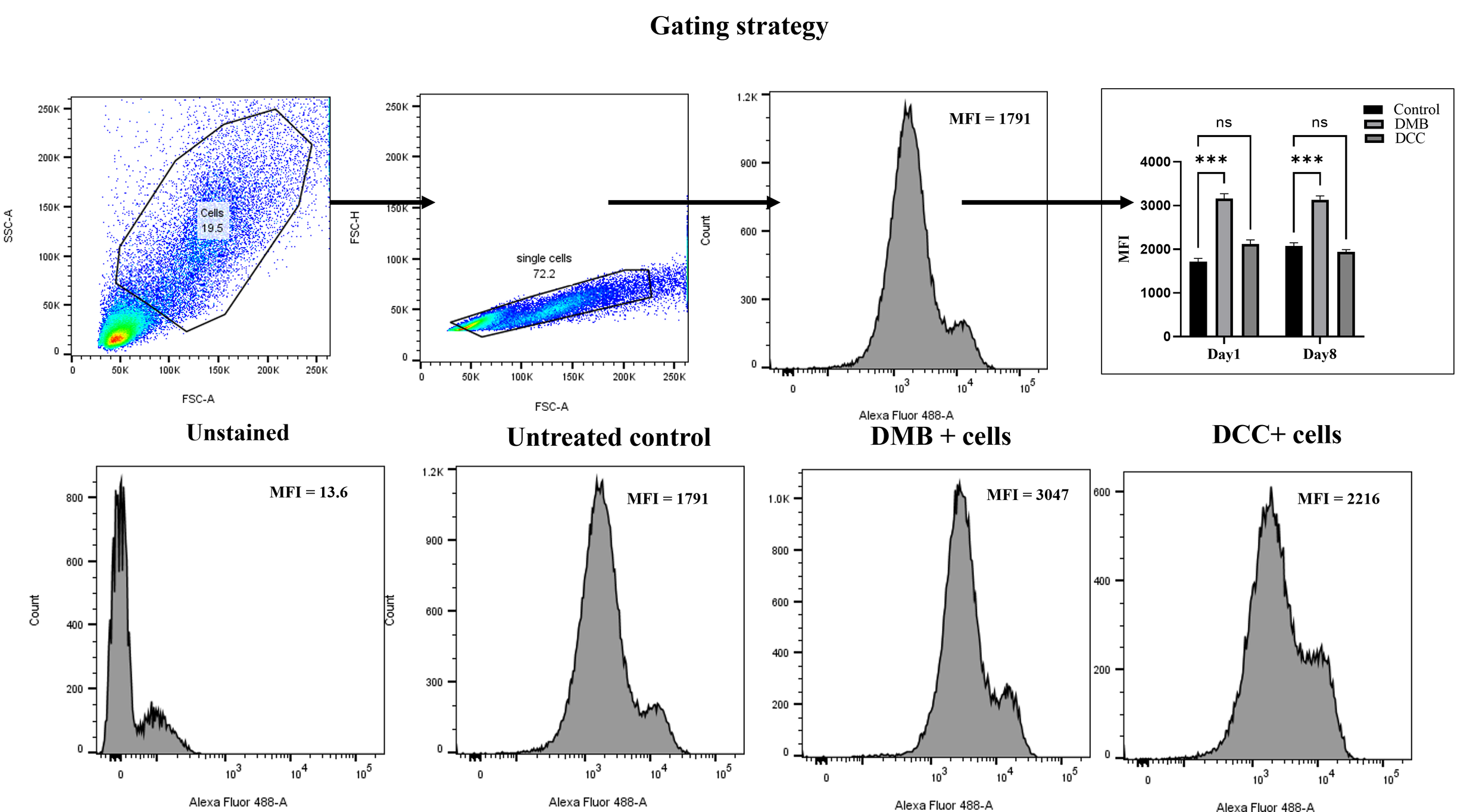

Supplement: S1 Fig — (a). Peripheral Blood Monocyte-derived Macrophages (PBMM) were gated based on forward and side scatter, (b) Doublet cells were excluded. (c) Representative histogram of reactive oxygen species expression in cells (d) analysis of results comparing the median fluorescent intensity (MFI) acquired from gated events among the study groups, (e) Representative histograms of ROS expression among unstained, untreated control, DMB and DCC treatments. (TIF) [file pone.0300331.s001.tif]

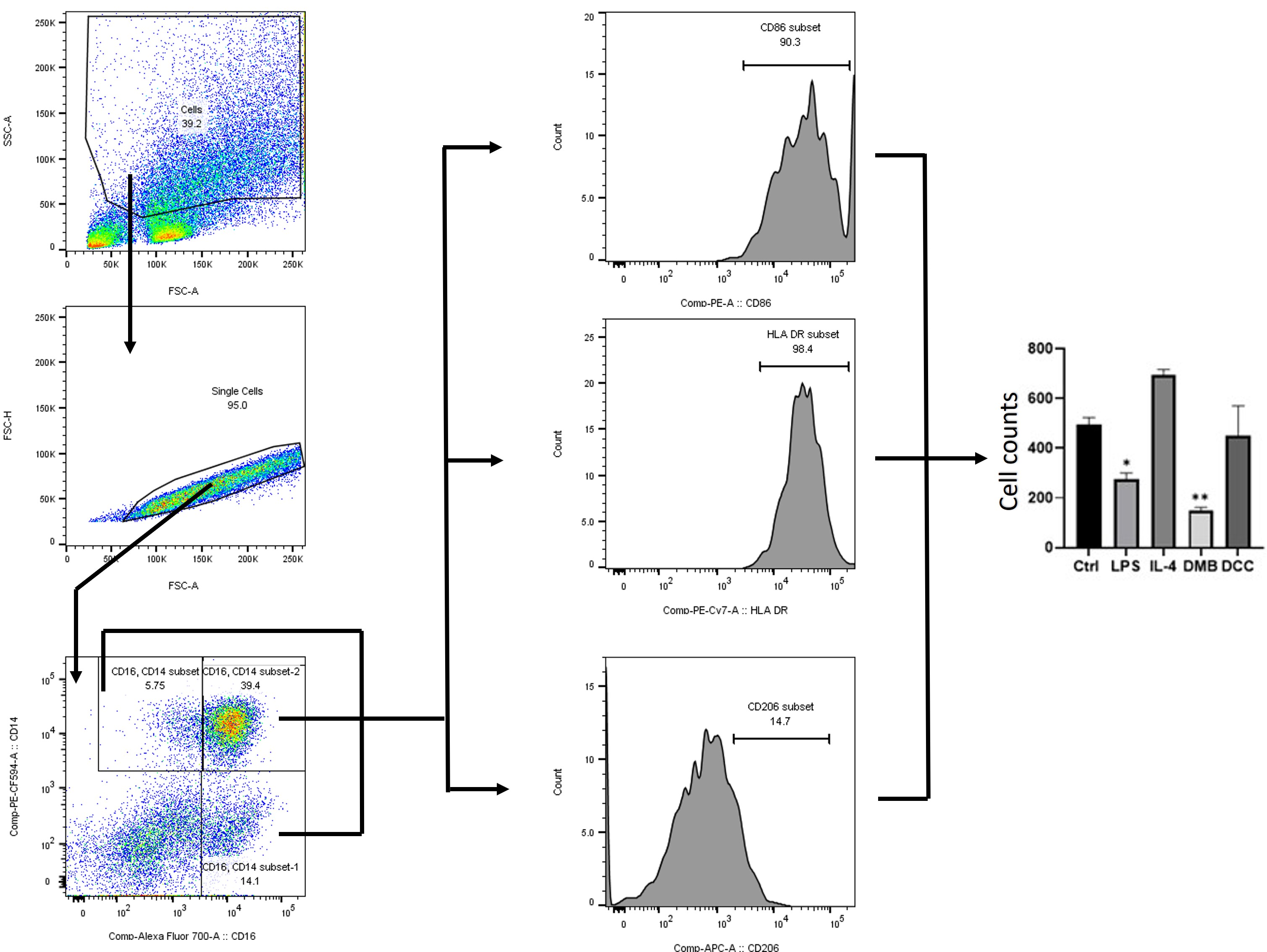

Supplement: S2 Fig — (a) Peripheral Blood Monocyte-derived Macrophages (PBMM) were gated based on forward and side scatter. (b) Doublet cells were excluded. (c) CD14 and CD16 subsets of macrophages were gated. (d) The different subpopulations of macrophages were determined based on the CD86, HLADR and CD206 gating followed by (e) analysis of results. (TIF) [file pone.0300331.s002.tif]
