# Supplementary material for: Response of human peripheral blood monocyte-derived macrophages (PBMM) to demineralized and decellularized bovine bone graft substitutes
Source: PLoS One. 2024 Apr 18;19(4):e0300331. doi: 10.1371/journal.pone.0300331 (PMC11025794; doi:10.1371/journal.pone.0300331)
Supplement: S1 Table — (DOCX) [file pone.0300331.s003.docx]

**Supplementary table 1: Details of antibodies used in flow cytometry analysis**

| **Antibody** | **Catalogue #** | **Company** | **Clone** | **Dilution** | **Fluorochrome** |
| --- | --- | --- | --- | --- | --- |
| CD14 | 562335 | BD | MφP9 (MφP-9) | 1/10 | PE-CF594 |
| CD16 | 557920 | BD | 3G8 (RUO) | 1/10 | Alexa Fluor 700 |
| CD86 | 557344 | BD | 2331 (FUN-1) | 1/5 | PE |
| CD206 | 550889 | BD | 19.2 (RUO) | 1/5 | APC |
| HLA-DR | 560651 | BD | G46-6 (L243) | 1/10 | PE-Cy^TM^7 |
